# Fermentation of Microalgal Biomass for Innovative Food Production

**DOI:** 10.3390/microorganisms10102069

**Published:** 2022-10-19

**Authors:** Cristiana Garofalo, Alessandra Norici, Lorenzo Mollo, Andrea Osimani, Lucia Aquilanti

**Affiliations:** 1 Dipartimento di Scienze Agrarie, Alimentari ed Ambientali, Università Politecnica delle Marche, via Brecce Bianche, 60131 Ancona, Italy; 2 Dipartimento di Scienze della Vita e dell’Ambiente, Università Politecnica delle Marche, via Brecce Bianche, 60131 Ancona, Italy

**Keywords:** *Arthrospira platensis*, *Chlorella vulgaris*, lactic acid bacteria, yeasts, dairy products, probiotics, functional foods, microalgae, fermentation, healthy diet

## Abstract

Fermentation is an ancient method used worldwide to process and preserve food while enhancing its nutraceutical profile. Alga-based fermented products have recently been developed and tested due to growing interest in healthy sustainable diets, which demands the development of innovative practices in food production, operating for both human health and Earth sustainability. Algae, particularly microalgae such as *Arthrospira platensis*, *Chlorella vulgaris*, and *Dunaliella salina*, are already cultivated as sources of food due to their valuable compounds, including proteins, pigments, lipids, carotenoids, polyunsaturated fatty acids, steroids, and vitamins. Due to their nutritional composition, functional diversity, and flexible metabolism, microalgae represent good fermentation substrates for lactic acid bacteria (LAB) and yeasts. This review presents an overview of the scientific studies on microalga fermentation underlining microalgae’s properties and health benefits coupled with the advantages of LAB and yeast fermentation. The potential applications of and future perspectives on such functional foods are discussed.

## 1. Introduction

Conventional food production is no longer sustainable and places a heavy burden on the environment as the global food system is causing greenhouse gas emissions, biodiversity loss, terrestrial ecosystem destruction, freshwater consumption, and nutrient loading [1]. Since the world’s population will reach almost 10 billion in 2050 [1,2], a current planetary challenge is feeding the global population while preserving natural ecosystems. Drastic changes are required to move towards healthy diets, regenerative production practices, and waste valorization. 

In the search for new or alternative crops to meet the growing demand for food (and feed), algae represent an emerging biological resource for sustainable innovative transformation [3,4]. Indeed, with respect to environmentally friendly food production, algae can be produced on non-arable land, allowing for a high rate of production per square meter compared to plants; intensive alga cultivation systems require only a minimal use of freshwater, and can even use seawater [5]. Food-processing wastewater also has the potential to be valorized as an algal growth medium once its biomass safety has been assessed [4].

### 1.1. Microalgae’s Properties and Health Benefits

Algae are a functional group of organisms able to use light as a source of energy to fuel cell metabolism and growth while releasing oxygen; they actually include species that are very phylogenetically distant, including eukaryotes and cyanobacteria, performing oxygenic photosynthesis [6]. 

Algae range in size from micrometers (for microalgae) to meters (for macroalgae or seaweeds). Microalgae are unicellular and belong to several taxonomic groups: Rhodophyta (red algae), Chlorophyta (green algae), Charophyta, Glaucophyta, Chlorarachniophyta, Euglenozoa (Euglenoids), Bacillariophyceae (Diatoms), Dinophyta (Dinoflagellate), Eustigmatophyceae, and Cryptophyta [7].

Moreover, in addition to oxygenic photosynthesis, many microalgae perform heterotrophic or mixotrophic metabolism. Such a unique ability, which cannot be found in plants [8,9], enables them to thrive in various extreme environmental conditions, even in the absence of light. Accordingly, microalgae are globally distributed and may be found in all types of environments, from deserts to arctic ice [10].

**Figure 1:**
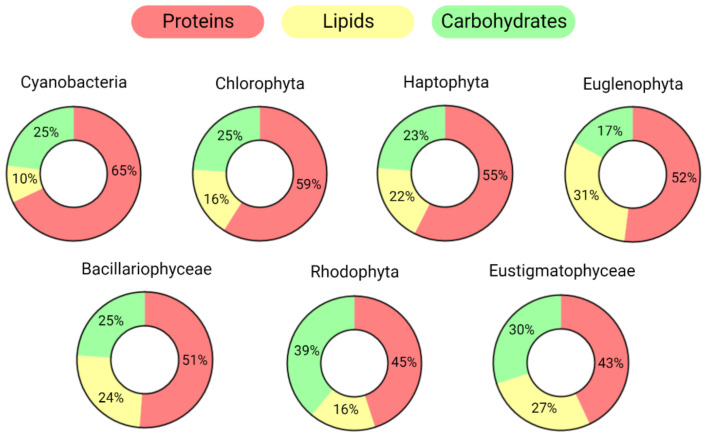
Average macromolecular compositions of the main microalgal taxonomic groups. Groups are ordered based on their protein abundance. Only the three main pools are reported: proteins, lipids, and carbohydrates. The values are normalized, excluding other main components of the dry weight such as ash, moisture, and pigments.

Due to their metabolic flexibility and diversity in terms of genetics, morphology, and habitat, the average macromolecular composition (regarding lipids, proteins, and carbohydrates) varies widely among distinct species. In nutrient-rich conditions, cyanobacteria usually accumulate higher concentrations of proteins than other phyla, while Rhodophyta and Euglenophyta store more carbohydrates and lipids, respectively (Figure 1).

The values reported in Figure 1 were calculated by averaging the organic compositions of species belonging to the same phylum/taxonomic group based on the scientific literature [3,11,12,13,14,15,16]; they refer to the composition determined during the exponential phase when extracellular nutrients were replete [17]. Both the phylogenetic history, which refers to the evolutive process of host and plastid endosymbiosis, and the environmental conditions are crucial in determining the macromolecular composition of algae [18,19].

Indeed, when nutrients become scant, algal acclimation strategies may lead to significant changes in the proportions of the main macromolecular pools [13,14,19]. 

**Figure 2:**
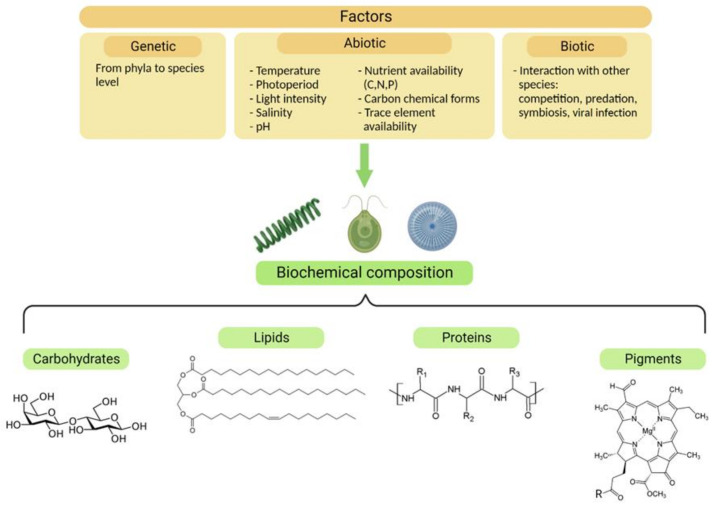
Factors influencing the biochemical composition of microalgae. Genetic, abiotic, and biotic factors can modify the concentrations and proportions of the four classes of molecules represented in the figure, which are desirable components for nutraceuticals.

Akin to nutrient starvation or limitation, many other abiotic factors contribute to the biochemistry of microalgae (Figure 2). Additionally, biotic factors such as competition for resources, predation, and symbiosis can drastically affect the behavior and composition of microalgae [18,20,21]. The presence of predators affects algal palatability, modulating their organic composition, and activating their chemical defenses [20].

From a biotechnological point of view, the easiest and most scalable way to modify the biochemistry of microalgae is through the manipulation of abiotic growth conditions, such as the light intensity and quality, photoperiod, pH, salinity, temperature, nutrient concentration, and chemical form [17,22]. Despite the consequent physiological and biochemical responses being species-specific, a common trend exists across microalgae: for instance, when nitrogen is not available, C-rich molecules, i.e., lipids and carbohydrates, accumulate inside the cell at the expense of the protein pool [13,16,17]. Usually, just one type of energy and C-storage molecular pool is preferred, since the precursor for fatty acid synthesis, glycerol-3-phosphate, is a catabolite derived from glucose [23,24], which is also the starting point for carbohydrate production. 

Carbohydrates play two main roles in algae: as structural components of the cell wall and as energy-storage compounds [11]. Glycogen, amylose, amylopectin, floridean starch, and chrysolaminarin are just a few of the storage compounds found in microalgae [14]. Lipids play similar roles in cells, and in addition to their concentrations, their biochemical properties are also modulated by abiotic factors. Light and temperature play major roles in the unsaturation rates of algal fatty acids [15,22]. In particular, low temperatures increase the degree of unsaturation of lipids and increase the percentages of PUFAs (polyunsaturated fatty acids) [25].

Among macromolecules, pigments play an essential role in microalgae, and, apart from chlorophyll *a*, which is the major photosynthetic pigment, many other accessory compounds play roles in light capture and protection from oxidative damage [12]. Widely used in aquaculture and medicine, β-carotene, astaxanthin, lutein, fucoxanthin, and phycobilins are only a few of the accessory pigments found in the algal plastids. Unlike chlorophylls, most of them are positively affected by irradiance [25]. Moreover, nitrogen-limited conditions may contribute to the accumulation of pigments such as astaxanthin and β-carotene in the green algae *Haematococcus pluvialis* and *Dunaliella salina*, respectively [25].

Microalgae are known to have been consumed by humans for thousands of years [3]; historical records report the consumption of the cyanobacteria *Nostoc*, *Aphanizomenon flos-aquae* (Klamath algae), and *Arthrospira* spp. (known commercially as Spirulina) [26]. The last is currently gathered when it naturally blooms in alkaline lakes by the Kanembu people in Chad and by the Myanmar people in the Republic of the Union of Myanmar [13], but archeological data also suggest that Aztecs commonly used Spirulina as a food during the 14th century [27].

Today, microalgal biomass is primarily sold for nutraceutical and food applications (Table 1). The microalgal species currently approved for EU and Italian markets are regulated and reported for the European Community by the Food Safety Regulation (EC 178/2002) and the Novel Food Regulation (EC 258/97; EC 2015/2283) [9,16,28]. Due to their high-quality nutritional protein value and beneficial amounts of fatty acids, vitamins, antioxidants, and many other bioactive molecules, microalgae are labeled with the nonscientific term “superfood” [3,26], and their health benefits are well recognized and documented [14,15]. As primary producers, algae are responsible for the accumulation of bioactive and bioavailable molecules in the entire food chain [27]. Thus, health benefits can be transferred to humans through the direct consumption of algae or indirectly by eating animals fed with algae; in aquaculture, algae are used as feed and supplements for fish farming [3]. 

Spirulina and *Chlorella* spp. are worthy of more detailed discussion, due to their widespread and historical use. The two main commercial species of Spirulina are *Arthrospira platensis* and *Arthrospira maxima*, which are filamentous, brackish cyanobacteria. Spirulina was found to have several properties useful for human health, including antioxidant and anti-hypertensive activity [29]. Moreover, the efficacy of Spirulina extract in combating foodborne pathogenic bacteria, either in vitro or in food matrices, has been demonstrated, suggesting perspectives for its further application as a new food preservative [30]. *Chlorella*, which includes *C. vulgaris* and *C. sorokiniana*, among others, is a unicellular eukaryotic spherical green alga belonging to the phylum Chlorophyta [31]. *Chlorella* has an appreciable content of arginine, a precursor involved in immune system functions, and sterols such as vitamin D2, involved in the absorption of calcium and phosphate [31].

Except for the aforementioned species, which are generally sold as dried whole biomass for the nutraceutical sector and formulated as tablets or powder, the algal market is dominated by high-value components extracted and purified from microalgae [9,27]. For instance, astaxanthin from *H. pluvialis* has antioxidant and anti-inflammatory effects, and its market price can reach USD 2000 per kg (total production of 300 tons per year) [3,14]. PUFAs (Ω3-6), mostly extracted from *Cryptothecodininum cohnii* and *Schizochytrium limacinum*, act at the cardiovascular and nervous system levels; their estimated value is USD 140 per kg [14]. 

Besides their beneficial properties, algae can also have adverse effects, which need to be considered. Some cyanobacteria can produce toxins under certain environmental conditions (e.g., microcystins, which can affect the nervous system). Contamination with toxic species may occur in algal cultivation systems for GRAS (generally recognized as safe) species [32]. Moreover, heavy metals (such as mercury, cadmium, and arsenic) can bioaccumulate inside algal cells. Additionally, the presence of a cell wall can restrict the access of human digestive enzymes to cell components, resulting in low biomass digestibility [13,14]. The cell wall is silicified in diatoms, calcified in some haptophytes, and organic in cyanobacteria and other eukaryotes [11]; therefore, in vitro digestion models are required to provide useful information about the nutrient bioavailability of microalgal organic matter [13].

**Table 1:** Bioactive compounds from microalgae, their applications, and their potential health benefits.

Microalgae	Product	Application	Effect on Human Health	Reference
*Arthrospira platensis (Spirulina)*	Biomass	Nutritional supplements and food ingredient	High protein content and rich in essential amino-acids. Rich in Fe, mineral elements, and vitamins	[33,34]
	Phycocyanin	Nutritional supplements	Antioxidant, anti-inflammatory	[35]
	EPS	Medical applications	Anti-thrombotic and anti-tumoral	[6]
*Aphanizomenon flos-aquae*	Biomass	Nutritional supplements	High protein content and essential fatty acids (Ω3)	[36]
*Chlorella vulgaris*	Biomass	Nutritional supplements and food ingredient	High protein and β-glucan content. Anti-inflammatory and anti-oxidant.	[37]
*Dunaliella salina*	β-carotene	Nutritional supplements and food ingredient	Antioxidant, pro-vitamin A, anti-allergic, anti-inflammatory	[38]
*Dunaliella tertioletca*	Vitamin A, B, E	Nutritional supplements	Maintenance of effective vision, protection against anemia and support of brain function, anti-oxidant	[39]
*Haematococcus pluvialis*	Astaxanthin	Nutritional supplements or supplements in aquaculture	Antioxidant, anti-inflammatory	[40]
*Isochrysis galbana*	EPA and DHA (Ω3)	Nutritional supplements or supplements in aquaculture	Anti-inflammatory, cardiovascular benefits, improves nervous system, atherosclerosis protection	[41,42,43]
*Cryptothecodininum cohnii*	DHA (Ω3)	Nutritional supplements or supplements in aquaculture	Cardiovascular benefits and improves nervous system	
*Schizochytrium* *limacinum*	DHA (Ω3)	Nutritional supplements or supplements in aquaculture	Cardiovascular benefits and improves nervous system	
*Nannochloropsis oceanica*	EPA (Ω3)	Nutritional supplements or supplements in aquaculture	Cardiovascular benefits and protection against atherosclerosis, anti-inflammatory	
*Porphyridium cruentum*	ARA (Ω6)	Nutritional supplements or supplements in aquaculture	Improve functional development in infants	[44]
*Porphyridium purpureum*	EPS	Medical application	Anti-thrombotic and anti-tumoral	[6]
*Phaeodactylum tricornutum*	Fucoxanthin	Medical application	Anti-tumoral and beneficial effect against obesity	[45]
*Euglena gracilis*	Paramylon	Medical application	Anti-inflammatory and anti-tumoral	[27]

EPS, exopolysaccharides; EPA, eicosapentaenoic acid; DHA, docosahexaenoic acid; ARA, arachidonic acid.

### 1.2. Advantages of Fermentation

Fermentation is an ancient method used worldwide for food processing and preservation [46,47,48,49]. From a biochemical point of view, fermentation has been defined as the breakdown of organic compounds by microorganisms, in either the presence (aerobic) or absence (anaerobic) of oxygen for energy production in terms of ATP production, coupled with other products that may be used as valuable commercial molecules within the food industry and other applications [46]. Alcoholic fermentation and lactic acid fermentation are the most common fermentation types. 

Alcoholic fermentation is a well-known process that is mainly carried out by yeasts that transform sugars into ethanol and CO_2_. *S. cerevisiae* is the main organism responsible for alcoholic fermentation, and it is frequently used as a starter culture in the transformation of sugar-rich matrices, such as grapes and other fruits or vegetables. To date, the alcoholic fermentation of algae by yeasts has been mainly applied in the biofuel industry [46].

Lactic acid fermentation is carried out by lactic acid bacteria (LAB), a group of Gram-positive, non-spore-forming bacteria that ferment carbohydrates as the main carbon source to produce lactic acid (homofermentation), or lactic acid along with ethanol or acetate and carbon dioxide (heterofermentation) as the major end-product [46,48]. LAB are widely used in the food industry due to their metabolic characteristics of being able to successfully transform and enhance the stability and quality of food products [47]. Indeed, the rapid acidification of food matrices is the main desired LAB activity; hydrolyzing activity, antagonistic activity, and biosynthetic activity are also present [49]. In addition to the production of lactic acid and other organic acids, LAB are able to promote the degradation of polymers such as proteins, lipids, and indigestible polysaccharides (e.g., starch, cellulose, hemicellulose, and others) by synthesizing enzymes that hydrolyze such polymers into smaller units, improving their digestibility and bioavailability [46]. These molecules may also act as flavor substances (as amino acids), thus influencing the aromatic profiles of food products, or show antioxidant, anti-inflammatory, antimicrobial, neuroprotective, anticoagulant, and immunomodulatory effects, thus enhancing the nutraceutical profiles of food [46]. It is worth noting that all the degradation and enzymatic processes that occur during fermentation are natural processes carried out by microorganisms without the use of solvents or acid–base hydrolyses; the latter are commonly applied for the extraction of bioactive molecules from complex matrices, causing the eventual production of large amounts of toxic wastes [46]. LAB are also able to excrete several metabolites that enhance food’s organoleptic and nutritional profiles [46,48]. Indeed, LAB may produce (i) functional molecules, such as gamma-aminobutyric acid (GABA), which is an important neurotransmitter in the nervous system and is involved in many human diseases; (ii) valuable nutritional compounds, such as polyphenols and polyunsaturated fatty acids; (iii) a wide range of organic acids with probiotic roles within the human intestine; (iv) antimicrobial molecules (e.g., bacteriocins) that increase the shelf life of foods; (v) antioxidant molecules, such as vitamins, polysaccharides, and phenolic substances; vi) exopolysaccharides (EPS) that increase the viscosity of foods; vii) a wide range of volatile compounds (e.g., organic acids, heterocyclic compounds, aldehydes, and ketones) that impact the aromatic profiles of a food; and viii) enzymes that reduce harmful and toxic molecules, such as mycotoxins and antinutritional factors, including phytic acid [46,48]. Overall, LAB’s properties and metabolism provide a wide range of marketable products differentiated in terms of their sensory, nutritional, and safety properties [49]. 

Moreover, some LAB strains can be added directly to food due to their probiotic functions, promoting human health. Indeed, probiotics are defined as “live microorganisms which when administered in adequate amounts, confer a health benefit on the host” [49]. Probiotics are resistant to the acidity of the gastrointestinal tract and to the bile environment; they are able to adhere to the intestinal mucosa by competing with pathogenic bacteria, improving the intestinal microbiota and producing health benefits, such as reducing cholesterol, inflammation, and lactose intolerance, as well as diarrhea [49]. 

The specific objective of this review is to provide an overview of microalgal fermentation for developing potential added-value food products.

## 2. Methodology

### 2.1. Search Procedure

The literature search was restricted to scientific publications dealing with microalga fermentation available in the PubMed (http://www.ncbi.nlm.nih.gov/pubmed accessed on 14 September 2022) and ScienceDirect (http://www.sciencedirect.com/ accessed on 14 September 2022) databases using the following keywords: ‘microalgae fermentation,’ ‘*Spirulina* fermentation,’ ‘*Chlorella* fermentation,’ ‘microalgae food fermentation,’ and ‘microalgae beverages fermentation.’ A cross-referencing approach was used to find other scientific studies. The inclusion criteria used for article selection were as follows: (i) publication in a peer-reviewed journal; (ii) published in English. The exclusion criteria were as follows: proceedings, project documents, theses, and papers on (a) microalgae’s nutritional aspects, (b) non-fermented foods based on microalgae, (c) microalga fermentation for purposes other than food production (e.g., cosmetics or environmental and bioenergetic applications), and (d) economic aspects related to algae.

The identified papers were first screened by title and abstract, and then, the full articles of the selected abstracts were retrieved and read. The literature search ultimately yielded 46 documents, including 5 reviews and 41 scientific articles. 

### 2.2. Database Generation

The following information was extracted from the 41 scientific articles: (i) the publication identification: authors, year of publication, and journal; (ii) the microalga species; (iii) the formulation and concentration of the microalga species; (iv) the microbial inoculum employed as the starter culture; (v) the fermentation conditions; (vi) the storage conditions for the novel fermented product, if applied; and (vii) the novel food product, if specified. 

## 3. Results and Discussion

### 3.1. Literature Review of Microalgal Fermentation for Novel Food Production

Table 2 shows a chronological distribution of the identified papers focused on the innovative application of microalgal fermentation in food production. In most of the papers, *Arthrospira* spp. (*A. platensis* or *A. maxima*) was chosen as a substrate for fermentation (35/41); two papers dealt with both *A. platensis* and *C. vulgaris*; in two papers, only *C. vulgaris* was the experimental organism; in one paper, *Pavlova lutheri* was used; and in one article, 10 different cyanobacterial and microalgal species, namely, *Nostochopsis lobatus*, *Nostoc commune*, *Nostoc flagelliforme*, *Nostoc verrucosum*, *A. platensis*, *Dunaliella tertiolecta*, *Chlorogonium* spp., *Porphyridium purpureum*, *Pleurochrysis carterae*, and *Euglena* spp., were selected for food fermentation.

According to Table 2, the microalgal powder/spray-dried/lyophilized/dry/dehydrated biomass was the most common microalgal formulation to be incorporated into the novel fermented food or beverage (33 out of 41 papers), while spirulina grains were reported in 1 paper, the use of fresh vs. oven-dried Spirulina biomass was compared in 1 paper, and the remaining 6 papers cited the microalgal biomass in filtrate/extract/wet/fresh form. The microalga concentration within each novel fermented product mainly ranged from 0.25% to 10%. Concerning the microbial inoculum used to ferment the microalgal species, most of the studies employed pure LAB cultures, mainly the yogurt starter cultures *Lactobacillus delbrueckii* spp. *bulgaricus* and *Streptococcus thermophilus*, and lattococci (*Lactococcus lactis* subsp. *lactis*, *Lactococcus lactis* subsp *cremoris*, and *Lactococcus casei*), as well as *Lacticaseibacillus casei* (basonym *Lactobacillus casei*), *Lactobacillus acidophilus*, *Lactiplantibacillus plantarum* (basonym *Lactobacillus plantarum*), *Lacticaseibacillus paracasei* (basonym *Lactobacillus paracasei*), *Levilactobacillus brevis* (basonym *Lactobacillus brevis*), *Lacticaseibacillus rhamnosus* (basonym *Lactobacillus rhamnosus*), *Lactobacillus helveticus*, *Streptococcus salivarius* subsp. *thermophilus*, *Enterococcus faecium*, *Weissella* spp., and *Leuconostoc* spp.; several strains of *Bacillus subtilis*, *Bacillus licheniformis*, and *Bacillus amyloliquefaciens* were also employed. Moreover, some probiotic cultures were also added to the novel formulation, including *Bifidobacterium lactis*, *Bifidobacterium animalis*, *Bifidobacterium* spp., and strains of *Lb. acidophilus* and *Lpb. plantarum*. Interestingly, two papers dealt with the use of a natural mixed starter culture, such as milk kefir grains and water kefir grains, while two studies developed novel products by fermentation with only pure yeast species, namely, *Debaryomyces hansenii*, *Kluyveromyces marxianus*, *Saccharomyces cerevisiae*, and *Hansenula polymorpha*. The scientific articles were then clustered according to topic: (i) 22 articles on the development and characterization of new fermented food products supplemented with microalgae, (ii) 13 articles on microalgae as the sole substrate for fermentation to obtain innovative functional foods or ingredients, and (iii) 6 articles on the effect of the addition of microalgae on the growth and viability of LAB and probiotics. These articles are listed in Table 2.

**Table 2:** Studies on microalgal fermentation for food applications.

Application	Microalga Species	Formulation	Concentration	Microbial Inoculum/Starter Cultures	Fermentation Conditions	Storage Conditions	Food Product	Reference
Microalgae as microbial growth promoter	*Arthrospira platensis*	Filtrate	1:1 filtrate added to synthetic media	*Lactococcus lactis* subsp. *lactis* C2, *Lactococcus casei* YK3, *Lactobacillus delbruekii* subsp. *bulgaricus* YL1, *Streptococcus salivarius* subsp. *thermophilus* TH4, *Lactobacillus* spp. JL2	37 °C, 24 h	n.d.	n.s.	[50]
	*Arthrospira platensis*	Extract	3 mg/mL in fermented milk	*Streptococcus thermophilus* TH4*, Lactococcus lactis* subsp. *lactis C2*, *Lactobacillus delbruekii* subsp. *bulgaricus* YL1, *Lactobacillus acidophilus* LO1	37 °C, 20 h	n.d.	Fermented milk	[51]
	*Arthrospira platensis*	Powder	3g/L in milk	*Lactobacillus acidophilus, Bifidobacterium* spp.*, Streptococcus thermophilus*	40 °C, 6 h	4 °C, 42 days, and 15 °C, 18 days	Fermented ABT milk	[52]
	*Arthrospira platensis; Chlorella vulgaris*	Spray-dried biomass	3 g/L in milk	*Lactiplantibacillus plantarum*, *Enterococcus faecium*	30 °C or 37 °C	n.d.	Fermented milk	[53]
	*Arthrospira platensis*	Powder	1, 5, 10 mg/mL	*Lactobacillus acidophilus* MTCC447, *Streptococcus thermophilus* MTCC1938, *Lacticaseibacillus casei* MTCC1423	37 °C, 10 h	n.d.	n.s.	[54]
	*Arthrospira platensis*	Powder	0.5, 1.0% (*w*/*v*) in milk	*Bifidobacterium animalis* subsp. *lactis* Bb12, *Lactobacillus acidophilus* La-5	42 °C, 6 h	5 ± 1 °C, 15 days	Fermented milks	[55]
Microalgae supplementation to fermented foods	*Arthrospira platensis*	Powder	0.5, 1.0% (*w*/*v*) in milk	*Streptococcus thermophylus*, *Lactobacillus delbrueckii* subsp. *bulgaricus*, *Lactobacillus acidophilus*	40 °C and 42 °C, until the pH 4.7 was reached	4 °C, 30 days	Yogurt and acidophilus milk	[56]
	*Arthrospira platensis; Chlorella vulgaris*	Powder	0.25, 0.5, 1.0% (*w*/*v*) in milk	*Lactobacillus acidophilus* LA-5, *Bifidobacterium lactis* BB-12, *Lactobacillus delbrueckii* subsp. *bulgaricus*, *Streptococcus thermophilus*	40 °C until the pH 4.7 was reached	5 °C, 28 days	Probiotic yogurt	[57]
	*Arthrospira platensis*	Powder	0.3, 0.5, 0.8% in milk	*Lactococcus lactis* subsp. *lactis*, *Lactococcus lactis* subsp. *cremoris*, *Lactobacillus acidophilus* LA-5	30 °C, 48 h	4 °C, 45 days	Probiotic feta cheese	[58]
	*Arthrospira platensis*	Powder	0.25, 0.5, 0.75, 1.0% (*w*/*v*) in milk	*Lactobacillus bulgaricus*, *Streptococcus thermophilus*	42 °C, 4 h	7, 14, 21, 28 days of refrigerated storage	Yogurt	[59]
	*Arthrospira platensis*	Grains	0.5, 1.0, 1.5%	*Lactobacillus delbrueckii* subsp. *bulgaricus*, *Streptococcus thermophilus*	n.r.	n.d.	Kareish cheese	[60]
	*Arthrospira platensis*	Dry biomass	1.0% (*w*/*v*) in soy milk	*Lactobacillus delbrueckii* subsp. *bulgaricus*, *Streptococcus thermophilus*	37 °C, 24 h	4 °C	Soy yogurt	[61]
	*Arthrospira platensis*	Dry biomass	0.5, 1.0, 1.5% in soy milk	*Lactobacillus delbrueckii* subsp. *bulgaricus, Streptococcus thermophilus*	37 °C, 16 h	4 °C	Soy yogurt	[62]
	*Arthrospira platensis*	Powder	5 mg/mL in milk	*Streptococcus salivarius* subsp. *thermophilus, Lactobacillus delbrueckii* subsp. *bulgaricus, Bifidobacterium* spp.	37 °C, 48 h	n.d.	Labneh	[63]
	*Arthrospira platensis*	Fresh vs oven-dried	0.1, 0.3, 0.5% (*w*/*v*) in milk	*Streptococcus thermophilus*, *Lactobacillus bulgaricus*	42 °C, 4 h	4 °C, 24 h	Yogurt	[64]
	*Arthrospira platensis*	Powder	0.5, 1.0, 1.5% (*w*/*w*)	*Lacticaseibacillus casei, Lactococcus lactis* subsp. *lactis, Lactococcus lactis* subsp. *cremoris*	40 °C until the pH 5.2 was reached	4 °C, 60 days	Feta-type cheese	[65]
	*Arthrospira platensis* SP6/CFTRI	Fresh wet biomass (spirulina milk emulsion)	10% (*w*/*w*)	*Lactobacillus delbrueckii* subsp. *bulgaricus*, *Streptococcus thermophilus*, *Lactobacillus acidophilus*	42 °C until the pH 4.6–4.7 was reached	6–8 °C	Probiotic yogurt	[66]
	*Arthrospira platensis*	Powder	0.25, 0.50, 1.0% (*w*/*v*) in milk	*Streptococcus thermophilus*, *Lactobacillus delbrueckii* subsp. *bulgaricus*, *Lactobacillus acidophilus*, *Bifidobacterium lactis*	40 °C until the pH 4.4 was reached (about 4 h)	4 ± 1 °C, 21 days	Ayran	[67,68]
	*Arthrospira platensis*	Powder	1.0% (*w*/*w*)	*Lactobacillus delbrueckii* subsp. *bulgaricus, Streptococcus thermophilus*	42 °C until the pH 4.6 was reached	4 °C	Low-fat yogurt	[69]
	*Arthrospira platensis* F&M-C256	Lyophilized biomass	10% (*w*/*v*) in vegetal soybean drink or water	*Lactiplantibacillus plantarum* ATCC 8014	37 °C, 72 h, 100 rpm stirring	n.d.	Vegetal soybean drink	[70]
	*Arthrospira platensis*	Dried biomass	n.s.	*Lacticaseibacillus paracasei*	40 °C until the pH 4.7 was reached	4 °C	Probiotic yogurt	[71]
	*Arthrospira platensis*	Algae biomass in water (5% w/v)	0.25, 0.50% (*w*/*v*) in skim milk powder (SSM) and in commercial soy-based beverages (SBB)	*Lactococcus lactis* subsp. *lactis*, *Lactococcus lactis* subsp. *cremoris*, *Lactococcus casei*, *Lactobacillus delbruekii* subsp. *bulgaricus*, *Streptococcus thermophilus*, *Lacticaseibacillus rhamnosus*, *Lactobacillus helveticus*, *Lactobacillus delbruekii* subsp. *lactis, Weissella* spp., *Leuconostoc* spp.	37 °C, 48 h	n.d.	Milk and soy fermented beverages	[72]
	*Arthrospira* spp.	Dry biomass	1.0, 2.0% (*w*/*v*) in milk	Milk kefir grains	25–37 °C, 24 h	n.d.	Milk kefir	[73]
	*Arthrospira platensis*	Powder	0.25, 0.50% in almond milk and soy milk	Lactobacilli and lactococci plant-based kefir culture	42 °C until a pH 4.5 ± 0.02 was reached	4 °C, 21 days	Vegan kefir (soy milk kefir and almond milk kefir)	[74]
	*Arthrospira platensis*	Powder	0.25, 0.5, 1.0 g/kg	Yogurt culture	20 °C, 24 h and then 12 °C, 48 h	n.d.	Greek soft cheese	[75]
	*Chlorella vulgaris*	Powder	1.5% (*w*/*v*) in soya drink	*Levilactobacillus brevis* ŁOCK 0944	30 °C, 4 h and then matured at 18 °C, 20 h	n.d.	Soya drink	[76]
	*Arthrospira* spp.	Lyophilized biomass	1.6 g, 2.4 g in 100 mL of distilled water	Water kefir grains	25 °C, 48 h	n.d.	Water kefir	[77]
Microalgae as the sole substrate for fermentation	*Pavlova lutheri*	Powder	1:15 (*w*/*v*)	*Hansenula polymorpha*	37 °C, 12 days	n.d.	n.s.	[78]
	*Arthrospira platensis*	Powder	2.0% (*w*/*v*)	*Lactiplantibacillus plantarum* B7, *Lactiplantibacillus plantarum* C8-1, *Lactiplantibacillus plantarum* 121, *Lactobacillus acidophilus* NCFM, *Bacillus subtilis* 168	37 °C, 24 h	n.d.	n.s.	[79]
	*Arthrospira maxima*	Powder	10% (*w*/*v*)	*Lactiplantibacillus plantarum* HY-08	37 °C, 4 days	n.d.	n.s.	[80]
	*Arthrospira maxima*	Powder	10% (*w*/*v*)	*Lactiplantibacillus plantarum* HY-08	37 °C	n.d.	n.s.	[81]
	*Arthrospira platensis*	Wet biomass	5 g in 30 mL of distilled water	*Lactiplantibacillus plantarum*	37 °C, 72 h in a shaker	n.d.	Fermented nutraceutical product	[47]
	*Arthrospira platensis* F&M-C256	Lyophilized biomass	10% (*w*/*v*)	*Lactiplantibacillus plantarum* ATCC 8014	37 °C, 72 h, 100 rpm stirring	n.d.	Probiotic-based products	[82]
	*Chlorella vulgaris*	Powder	0.1, 1.5% (*w*/*v*)	*Levilactobacillus brevis* ŁOCK 0944, *Levilactobacillus brevis* ŁOCK 0980, *Levilactobacillus brevis* ŁOCK 0992, *Levilactobacillus brevis* MG451814	30 °C, 24 h	n.d.	n.s.	[83]
	*Arthrospira platensis*	Powder	2.0% (*w*/*v*)	*Lactiplantibacillus plantarum* DY-1, *Lacticaseibacillus casei* KDB-LC, *Lactobacillus acidophilus* KDB-03, *Lactobacillus acidophilus* KDB-08, *Bacillus subtilis* ND, *Bacillus* spp., *Bacillus amyloliquefaciens* LXZ	37 °C, 72 h	n.d.	n.s.	[84]
	*Arthrospira platensis*	Dehydrated biomass	n.s.	*Lacticaseibacillus casei* 2240, *Lacticaseibacillus rhamnosus* GG	37 °C, 48 h	n.d.	New fermented food supplements	[85]
	*Nostochopsis lobatus*, *Nostoc commune, Nostoc flagelliforme*, *Nostoc verrucosum*, *Arthrospira platensis*, *Dunaliella tertiolecta*, *Chlorogonium* spp., *Porphyridium purpureum*, *Pleurochrysis carterae*, *Euglena* spp.	Powder	10% (*w*/*v*)	*Lactococcus lactis* subsp. *lactis*, *Lactiplantibacillus plantarum*	37 °C, 72 h	n.d.	n.s.	[86]
	*Arthrospira platensis*	Fresh	10 g with 10 mL of physiological solution	*Lactiplantibacillus plantarum*	30°C, 72 h	n.d.	n.s.	[87]
	*Arthrospira platensis*	Powder	4.0% (*w*/*v*)	*Debaryomyces hansenii*, *Kluyveromyces marxianus*, *Saccharomyces cerevisiae*	28 °C, 48 h, 130 rpm stirring	n.d.	n.s.	[88]
	*Arthrospira platensis*	Dried powder	4.0% (*w*/*v*)	*Lactobacillus helveticus* B-4526, *Lactiplantibacillus* *plantarum* B531, *Lacticaseibacillus rhamnosus* B-442, *Lacticaseibacillus casei* B-1922, *Bacillus subtilis* B-3384, *Bacillus subtilis* B-3387, *Bacillus licheniformis* NRS-1264	LAB strains at 37°C, 48 h, 100 rpm stirring; *Bacillus* strains at 28°C, 48 h, 130 rpm stirring	n.d.	n.s.	[89]

n.d., not determined; n.s., not specified; *w/v*, weight/volume; *w/w*, weight/weight; rpm, revolutions per minute.

### 3.2. Effect of the Addition of Microalgae on Growth and Viability of LAB and Probiotics

The study of Parada et al. [50] was the first to explore the feasibility of the fermentation of *A. platensis* filtrate using several LAB species added to a synthetic medium, and a stimulatory effect on LAB growth was observed (Table 2). These authors hypothesized the presence of extracellular products released by *A. platensis* within the medium that promoted LAB growth in vitro. Subsequently, the studies of de Caire et al. [51], Varga et al. [52], Gyenis et al. [53], and Mocanu et al. [55] tested the potential of *A. platensis* and *C. vulgaris* as bacterial growth promoters in milk and confirmed that the algae boosted the growth and survival of several LAB and probiotic strains: *Str. thermophilus*, *Lc. lactis* subsp. *lactis*, *Lb. delbruekii* subsp. *bulgaricus*, *Lb. acidophilus*, *Bifidobacterium* spp., *Lpb. plantarum, E. faecium*, and *Bifidobacterium animalis* subsp. *lactis*. Their results paved the way for research on the development of innovative microalga-based dairy products. In addition to stimulating LAB growth, *A. platensis* biomass was also able to inhibit some human pathogenic bacteria, such as *Escherichia coli*, *Pseudomonas aeruginosa*, *Proteus vulgaris*, *Staphylococcus aureus*, *B. subtilis*, and *Bacillus pumulis,* in an in vitro agar well diffusion assay [54]. It has been hypothesized that the antimicrobial activity shown by *A. platensis* was due to some intracellular or extracellular biologically active substances produced as secondary metabolites [30,54].

Overall, these studies confirmed the untapped potential of *A. platensis* and *C. vulgaris* in acting as prebiotic factors in fermented foods.

### 3.3. Development and Characterization of New Fermented Food Products Supplemented with Microalgae

Most of the existing scientific studies on algal fermentation have used macroalgae or seaweeds [46,90], but microalgal fermentation may expand the applications of algae in the food industry. The enrichment of fermented dairy products with microalgae as suppliers of bioactive compounds is considered an interesting route towards the development of sustainable and healthier dairy products that are also sources of beneficial microorganisms [91,92]. To the best of the authors’ knowledge, *Arthrospira* spp. and *C. vulgaris* are the two main species applied for fermented food fortification (Table 2). The analyzed studies were mainly focused on the production of novel dairy products, such as milk-based fermented beverages (Ayran, Labneh, fermented milks, acidophilus milks, milk kefir, yogurt, and probiotic yogurt) and different cheeses (probiotic Feta-type cheese, Kareish cheese, and Greek soft cheese), as well as lactose-free beverages, including soy yogurt and soy fermented beverages, vegan kefir, and water kefir (Table 2). In detail, the studies on ayran [67,68] confirmed the positive influence of *A. platensis* incorporation on the growth and survival of *Str. thermophilus*, *Lb. delbrueckii* spp. *bulgaricus*, *Lb. acidophilus*, and *Bifidobacterium lactis* due to the high amounts of nitrogenous materials (such as proteins, free amino acids, peptone, and peptides), minerals, B vitamins, EPS, adenine, hypoxanthine, and all the organic and inorganic nutrients that acted as LAB growth promoters and were obtained from Spirulina. However, as also demonstrated for probiotic yogurt, while Spirulina promotes LAB growth, it may impair the rheological properties of the final product. For example, in Ayran, as well as in a probiotic yogurt, a decrease in viscosity with an increasing amount of Spirulina was observed, probably due to the high LAB load and titratable acidity level, which enhanced casein proteolysis [67,68,71]. Studies on Ayran enriched with Spirulina also showed that an algal concentration higher than 0.25% decreased taste and thickness scores [67,68]. Abbas et al. [63] reported similar behavior regarding the viability of probiotic bacteria, but demonstrated that the addition of 5 mg/mL of *A. platensis* powder in milk is the recommended formulation for preparing an acceptable product, in terms of taste and color, called Labneh (a probiotic concentrated-yogurt from the Middle East). By contrast, the studies of Guldas and Irkin [56], Beheshtipour et al. [57], and Barkallah et al. [59] demonstrated that the addition of 0.25–0.5% of Spirulina powder to milk for yogurt production was sufficient to promote the fermentation process and to develop a product with acceptable sensory and rheological properties, as well as nutraceutical properties. Spirulina is a good source of molecules with antioxidant activity, including carotenoids, phycocyanin, chlorophylls, and natural flavoring and coloring agents. Moreover, Spirulina serves as a carrier of proteins and dietary fibers that play an important role in maintaining the product’s texture, acting as a physical stabilizer [59]. Additionally, the study of Patel et al. [66] aimed at developing a probiotic yogurt enriched with carotenoids by adding fresh Spirulina biomass. As underlined by the study, upon homogenization for complete biomass dispersion, up to 7% of the algal biomass was efficiently incorporated into the milk, avoiding the non-uniform distribution and unpleasant sensory attributes that may occur when Spirulina powder is used. Accordingly, Bchir et al. [64] reported that the addition of 0.3% of fresh Spirulina resulted in a yogurt with enhanced nutritional value and an apparent viscosity with higher organoleptic acceptability with respect to that obtained with dried Spirulina biomass. 

To the authors’ knowledge, the study of Laela et al. [73] represents one of the very few studies that has employed mixed natural starter cultures, such as kefir grains, instead of pure cultures to produce Spirulina-based fermented products (Table 2). Kefir grains are composed of a mixture of LAB, yeasts, and sometimes acetic acid bacteria that coexist in a symbiotic relationship embedded within a matrix of EPS and proteins of microbial origin [93,94]. Due to this complex and unique microbial community, kefir is a popular drink rich in bioactive compounds such as peptides, amino acids, bacteriocins, folic acid, calcium, and vitamins, and several health benefits are linked to its daily consumption [93,94]. Milk supplemented with Spirulina fermented in the presence of kefir grains resulted in a functional kefir–Spirulina beverage with increased nutritional value and the capacity to control glycemic status; it enhanced the antioxidant status in a diabetic rat model, thus showing its potential for use in the human diet to manage diabetes [73]. 

Concerning cheeses, the two studies on Feta cheese demonstrated a positive effect of Spirulina in stimulating bacterial growth and viability, even during prolonged storage, as well as beneficial results in terms of the iron and protein amounts and the texture of the final product [58,65]. The Greek soft cheese produced in the presence of Spirulina was characterized by an increased protein content, a good microbiological profile, and an acceptable sensory profile, which was preferred by panelists when Spirulina was added at 0.25–0.5% *w*/*w*, due to a reduction in the typical flavor of the algae [75]. Intriguingly, Kareish cheese was enriched with small grains instead of Spirulina powder, and this solution was preferred by the panelists, since the product mimicked Roquefort cheese [60]. Fortification with Spirulina increased the nutritional value (in terms of protein, fat, ash, acidity, iron, total phenols, total flavonoids, and carotenoid contents), improved the antioxidant activity, and enhanced the texture profile of the cheese [60].

Besides fermented dairy products, a few studies have explored the development of vegetal alternatives to milk-based products fortified with microalgae. In detail, soy yogurt [61,62], soy milk or soya drink [70,72,74,76], almond milk [74], and plain water [70,77] were supplemented with *A. platensis* or *C. vulgaris*. The selected studies confirmed that the products based on Spirulina or *C. vulgaris* ingredients had added value and further supported the hypothesis that microalgae boost LAB metabolic activity (i.e., by increasing lactic acid production) and viability [70,72,74,76]. In detail, it has been observed that the total phenolic content, as well as the promoting effect on LAB growth during cold storage, increased in vegan kefir with an increase in the concentration of *A. platensis* [74]. Similarly, Niccolai et al. [70] showed an increase in the total phenolic content and antioxidant activity of a soybean drink upon its enrichment with *A*. *platensis* once it was fermented by the probiotic bacterium *Lpb. plantarum* ATCC 8014. The strong intracellular antioxidant activity that was observed in this drink has been attributed to the antioxidant components (e.g., phenols and phycocyanin) released by the cyanobacterial biomass during fermentation, acting in a synergistic manner with the bacterial culture. The in vitro digestibility of the soybean drink and water with added Spirulina was also tested. A significant improvement in digestibility was observed for water supplemented with Spirulina after 72 h of fermentation, while no effect of fermentation was found for the enriched soybean drink [70]. These data are in agreement with the study of Niccolai et al. [82], who observed a slight increase in digestibility in a fermented Spirulina extract. The authors hypothesized that the high number of bacterial cells at the end of the fermentation could have lowered the final digestibility of the products [70,82]. To the authors’ knowledge, only a few studies have focused on the in vitro digestibility of fermented microalgae, thus suggesting that this issue warrants further investigation.

Moreover, by using a gastrointestinal tract simulator, Scieszka et al. [76] demonstrated the effectiveness of a soya drink supplemented with *C. vulgaris* in increasing the survival rate of *Lev. brevis* ŁOCK 0944. They proposed the development of a new functional, plant-based, lactose-free fermented probiotic product. Accordingly, Sengupta et al. [61] proposed a soy yogurt fortified with *S. platensis* as a functional food due to its in vivo therapeutic effect on hypercholesteremic cardiovascular disease mediated by lowering cholesterol, improving serum HDL-C, and increasing hepatic antioxidant enzymes, with positive effects on liver function. Finally, Spirulina biomass was also used in water kefir fermentation to replace sugar [77]. As expected, the addition of Spirulina increased the nutritional profile of the water kefir in terms of the protein content, but, intriguingly, the fermentation process carried out by water kefir grains further improved the protein content with respect to the content in the unfermented samples. Moreover, a range of various saturated, unsaturated, and polyunsaturated fatty acids that are beneficial for health were detected after fermentation, thus confirming the significant contribution of kefir grains’ microbiota to the overall quality of the final product [77]. Notably, Martelli et al. [72] showed that Spirulina’s boosting effect on LAB growth and fermentation is variable depending on the LAB cultures, substrate, and Spirulina concentration; therefore, further research is required for the technological optimization of a specific fermentation process. The maximum boosting effect on LAB growth was obtained with the use of 0.25% Spirulina wet biomass, while a higher Spirulina concentration exhibited fluctuating effects on LAB growth.

### 3.4. Fermented Microalgae as Innovative Functional Foods or Ingredients 

Some studies specifically focused on the evaluation of using microalgal biomass or extract as the sole substrate for LAB or yeast fermentation in order to obtain new functional foods or supplement formulations (Table 2). Overall, these studies aimed to test how the microbial fermentation changed the nutritional composition, sensory attributes, and functional properties of microalgae. LAB cultures were able to hydrolyze the cell wall polymers of microalgae into simpler compounds via their metabolic activities, producing molecules with nutraceutical properties [46,47]. Moreover, Spirulina wet biomass was fermented by *Lpb. plantarum*, resulting in a higher content of total phenolic compounds, C-phycocyanin, and free methionine, as well as improved DPPH-radical-scavenging capacity, ferric reducing antioxidant power, oxygen radical absorbance capacity, and protein fragmentation, with the release of bioactive peptides, compared to the results obtained for the unfermented sample, following 72 h of fermentation [47]. Niccolai et al. [82] reported that *A. platensis* was a suitable growth substrate for the probiotic bacterium *Lpb. plantarum* ATCC 8014, and the fermentation product showed a marked increase in antioxidant activity and total phenolic content (79% and 320%, respectively) with respect to the values in the control samples. Accordingly, the study of Jamnik et al. [87] confirmed a higher cellular antioxidant capacity and total phenolic content for biomass of *A. platensis* fermented by *Lpb. plantarum* compared to those observed in the unfermented control. Furthermore, a maximum LAB count, higher lactic acid concentration, and a drop in pH were recorded in the first 24 h of fermentation. The low pH and the absence of pathogenic bacteria also suggested that fermented *A. platensis* may be included in new food formulations to promote microbial stability. Additionally, the non-protein nitrogen level increased, indicating higher protein degradation and bioavailability and fat content reduction in comparison with the unfermented control. Intriguingly, the microalga *P. lutheri* also showed high antioxidant activity after fermentation with the yeasts *H. polymorpha*, and it has been suggested as a potential source of natural antioxidants [78]. Furthermore, 12 strains from 10 species of microalgae and cyanobacteria—*N. lobatus*, *N. commune*, *N. flagelliforme*, *N. verrucosum*, *A. platensis*, *D. tertiolecta*, *Chlorogonium* spp., *P. purpureum*, *P. carterae*, and *Euglena* spp.—have been assayed under fermentation with *Lc. lactis* subsp. *lactis* and *Lpb. plantarum* [86]. Not all the species were able to support the LAB fermentation, and only the fermented aqueous extracts of wild *N. commune* and sake lees cultured *Euglena* spp. fermented with *Lc. lactis* were found to be promising in terms of O_2_—radical-scavenging capacity and anti-glycation activity. 

Other studies aimed to demonstrate the neuroprotective effect and the memory-enhancing activity of *A. maxima* biomass fermented by a *Lpb. plantarum* strain isolated from fermented vegetables [80,81]. The fermentation process was combined with the ultrasonic extraction of β-carotene at 40 kHz for 4 h [80]. The high antioxidant capacity of this extract was attributed to the high β-carotene content obtained and to other biologically active substances produced by LAB fermentation, which were able to strongly enhance the brain-derived neurotrophic factor (BDNF)/p-CREB signaling pathways that prevent dementia in mice induced by oxidative stress. 

Recently, the study of Ścieszka and Klewicka [83] examined the effect of *C. vulgaris* on the growth kinetics, acidifying activity, proportion of lactic acid isomers, and enzymatic profiles of four strains of *Lev. brevis*. Notably, these LAB strains accelerated their growth by shortening the logarithmic phase when cultured in synthetic media containing *C. vulgaris*, and the growth rate is a crucial parameter from a technological point of view. In parallel, the LAB acidifying activity increased, thus reflecting a potential improvement in the LAB antagonistic activity against the growth of pathogenic bacteria. Moreover, the amount of L-lactic acid increased relative to that of D-lactic acid, another advantageous feature for food exploitation. Finally, the β-glucosidase and leucine arylamidase activities were enhanced in all the strains, and high activities of valine arylamidase, α-galactosidase, and α-glucosidase were also detected in one LAB strain. The cited enzymatic activities play an important role in food production, since they convert complex molecules into antioxidants and flavor compounds. 

Finally, several studies aimed to investigate the influence of different microbial species and treatments on the sensory properties of fermented Spirulina. Despite the well-known nutritional properties of algae, their use in food is limited due to their unique aroma, taste, flavor, and color attributes [88]. The study of Sahin et al. [88] successfully showed a reduction in the typical aromas of Spirulina, such as “seaweed,” “umami,” “cardboard,” “earthy/muddy,” and “cereal,” by testing the fermentation performance of three pure cultures of yeasts: *D. hansenii*, *K. marxianus*, and *S. cerevisiae.* The three yeasts were able to grow in the sole presence of spirulina, and the *K. marxianus*-fermented spirulina was characterized by “fermented” and “rose” attributes. *K. marxianus* also exhibited the strongest ability regarding protein hydrolysis; the amounts of glutamic acid, methionine, lysine, isoleucine, and valine in the final product were all increased. Martelli et al. [85] evaluated the LAB fermentation of sterilized Spirulina biomass based on the volatile organic compound (VOC) profile. The sterilization of the matrix before the fermentation process was the best treatment for improving the final aroma of the product, reducing off-flavors and ensuring that it was safe for consumers. Bao et al. [79] applied different LAB strains and a *B. subtilis* culture to Spirulina biomass to improve the volatile component profile, to reduce off-flavors through deodorization, and to hydrolyze the protein. The mixed fermentation of spirulina by *Lpb. plantarum* and *B. subtilis* efficiently produced a pleasing aroma in the final product, since most of the undesirable aromatic notes associated with spirulina were removed, while other volatile molecules, such as acetoin (responsible for a creamy flavor), ethyl L(-)-lactate, lactic acid, and (R,R)-2,3-butanediol were produced via the fermentation. Furthermore, high protein bioavailability was observed: more than 30% of the protein was converted into polypeptides and amino acids. Similarly, Yu et al. [84] tested several LAB and *Bacillus* strains, including in combination, to investigate the effects on the components and bioactivity of Spirulina. The study confirmed the efficacy of all of the strains in enhancing the protein content, the content of amino acids, and the ratio of essential amino acids to total amino acids, and the LAB and *Bacillus* strain mixture showed the best effect. The fermentation also affected the VOC profile, thus mitigating the unpleasant odor of spirulina. Finally, the antioxidant and antibacterial activity of the fermented Spirulina product was also confirmed. Very recently, Kurt et al. [89] confirmed the positive influence of four different LAB and three different *Bacillus* strains on spirulina biomass transformation in terms of the VOCs and sensory properties, as well as total and free amino acids and protein hydrolysis. LAB strains were responsible for the highest proteolytic activity and highest sensory attribute score, while the *Bacillus* strains increased the total amino acids and total essential amino acids. 

**Figure 3:**
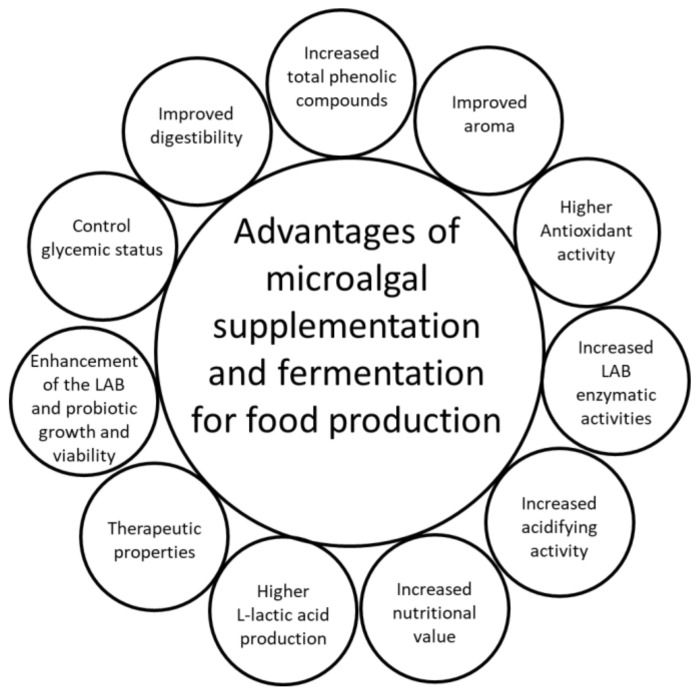
Advantages of microalgal supplementation and fermentation for potential use in innovative food production; LAB, lactic acid bacteria.

The beneficial effects of microalgal supplementation and fermentation for potential use in innovative food production are summarized in Figure 3.

## 4. Conclusions and Future Perspectives

The increasing growth of the population, in conjunction with reduced food resources, has resulted in increasing global demand for sustainable protein sources and functional foods. Microalga-based products are recognized as a promising solution for reducing global hunger and mitigating climate change [95]. According to the present review, microalgal fermentation driven by microorganisms may represent a safe and cheap technology for increasing the nutritional value, digestibility, and acceptability of microalgae. Indeed, based on the literature, microalgae are able to support and enhance pure microbial cultures, mixed cultures, and probiotic growth without the supplementation of other carbon sources, as well as through the enrichment of synthetic media, milk, or vegetal-based substrates with macro- and micronutrients. Fermentation has been proved to increase the bioactive profiles of microalgae by (i) reducing lipids, (ii) increasing the protein content, (iii) increasing the total phenolic content, (iv) increasing the pigment content (i.e., phycocyanin), (v) promoting the antioxidant activity, (vi) releasing free amino acids and bioactive peptides, (vii) increasing protein bioavailability, (viii) affecting the fatty acid composition, and (ix) producing functional metabolites. 

Among the few microalgae tested in research trials to date, *A. platensis* has the potential to be exploited as a suitable substrate for microbial growth and fermentation to produce valuable fermented foods and beverages from nutritional, nutraceutical, and economic points of view. The fermented microalga field is in its infancy and has been only partially explored; the results obtained for spirulina are encouraging and pave the way for further studies on other microalgal species. Being able to manipulate the composition of microalgae may also be crucial for optimizing the fermentation process. Further studies to identify and purify the specific bioactive compounds obtained from microalgal fermentation that play key roles in many biological activities are needed.

Besides the potential health benefits, an overall reduction in the sensory acceptability of fermented products is often reported, especially when microalgae are added at high percentages. Future studies to improve the sensory attributes of such products are needed in order to meet the consumer demand for food that is both nutritious and pleasant. Moreover, the variability of LAB growth within fermentation mixtures due to several factors (e.g., the microbial strains and natural starter cultures, substrates, and microalga concentrations) is another area that needs to be researched in greater depth in order to guarantee the optimization and reproducibility of the fermentation process as required by the food industry.

## Data Availability

Not applicable.
